# Special Issue “COVID-19 Coagulopathy: Advances on Pathophysiology and Therapies”

**DOI:** 10.3390/ijms25063548

**Published:** 2024-03-21

**Authors:** Eliza Russu, Emil-Marian Arbănaşi, Alexandru Șchiopu

**Affiliations:** 1 Clinic of Vascular Surgery, Mures County Emergency Hospital, 540136 Targu Mures, Romania; eliza.russu@umfst.ro; 2 Department of Vascular Surgery, George Emil Palade University of Medicine, Pharmacy, Science and Technology of Targu Mures, 540139 Targu Mures, Romania; 3 Doctoral School of Medicine and Pharmacy, George Emil Palade University of Medicine, Pharmacy, Science and Technology of Targu Mures, 540142 Targu Mures, Romania; 4 Centre for Advanced Medical and Pharmaceutical Research (CCAMF), George Emil Palade University of Medicine, Pharmacy, Science, and Technology of Targu Mures, 540139 Targu Mures, Romania; 5 Department of Translational Medicine, Lund University, 22100 Lund, Sweden; alexandru.schiopu@med.lu.se; 6 Department of Internal Medicine, Skåne University Hospital Lund, 22185 Lund, Sweden; 7 Nicolae Simionescu Institute of Cellular Biology and Pathology, 050568 Bucharest, Romania

The Special Issue on COVID-19 coagulopathy initiated one year ago aimed to shed light on the mechanisms underlying the changes in the coagulation status making SARS-CoV-2 infection such a tough adversary for every one of the medical specialties encountering it, along with overseeing the therapeutic applications derived from the current understanding of these mechanisms.

Every emergency and ICU compartment admitting critical patients during the SARS-CoV-2 pandemic fought against the clinical manifestations of arterial and venous thrombosis, along with a variety of severe disorders affecting almost all organs and systems [1,2,3,4,5].

Bacterial infections are associated with disseminated intravascular coagulation (DIC), but COVID-19 coagulopathy differs from DIC occurring during severe bacterial infections and sepsis. In sepsis-associated DIC, disease severity and mortality were associated with a low platelet count and platelet time (PT) prolongation, whereas severe COVID-19 coagulopathy was best reflected by increased levels of D-dimers. Laboratory, clinical, and histopathologic findings suggested that COVID-19 coagulopathy is characterized by dysregulated hemostasis, leading to the formation and degradation of micro- and macrovascular thrombi [6]. There appears to be a close link between severe systemic inflammation and dysregulated hemostasis in the pathophysiology of the disease [7]. COVID-19-related coagulopathy is also associated with thrombocytopenia. In a meta-analysis of 7613 COVID-19 patients, Julien Maquet et al. [8] have shown that thrombocytopenia was worse in the critically ill group compared to patients with mild forms of the disease.

The “two-path unifying theory” of hemostasis and endotheliopathy aimed to explain the imbalance between coagulation and inflammation. Viral protein S adhesion to endothelial membrane angiotensin-converting enzyme (mACE2) is a widely recognized pathway for viral penetration into the cells [9]. ACE-2 polymorphisms, alongside gender, race and age differences, are the major factors contributing to the wide variability in COVID-19 deaths, as Wooster et al. [10], Santosh et al. [11], and Srivastava et al. [12] have shown in their reports.

The main role of ACE-2 is the degradation of angiotensin II at the endothelial surface, counteracting its potent vasoconstrictor and pro-inflammatory effects [13]. Experimental studies have shown how the injection of the recombinant SARS spike protein led to elevated levels of angiotensin II in mice, possibly via the downregulation of ACE-2 expression on the endothelium. In turn, treatment of the mice with angiotensin II receptor type 1 (AT1R) blockers reduced disease severity [14]. Thus, understanding the role of the interplay between ACE-2, ACE, and their receptors in the pathogenesis of COVID-19 is very important. The downregulation of endothelial ACE2 as a consequence of SARS-CoV-2 infection inhibits the vasodilator, anti-inflammatory, and anti-coagulant effects of the enzyme, leading to endothelial dysfunction, vasoconstriction and a prothrombotic status. Recombinant human ACE-2 has been tested as a potential therapy for acute lung injury, as it may act as a decoy receptor for SARS-CoV-2 in the circulation and prevent the binding of the virus to the endothelium [15,16].

The virus invades type II pulmonary alveolar and endothelial cells, releasing danger-associated molecular patterns (DAMPs), proinflammatory cytokines, and chemokines. The subsequent activation of leukocytes and platelets leads to a cytokine storm, characterized by a potent release of interleukin-1, -6, -8, -10, and -12 (IL-1, IL-6, IL-8, IL-10, Il-12), tumor necrosis factor-alpha (TNF-α), interferon-γ, C–X–C motif chemokine 10, and monocyte chemoattractant protein-1 [7]. These inflammatory mediators trigger the further recruitment and activation of leukocytes and platelets, activation of the coagulation cascade, and generation of intravascular thrombin in a continuous loop [7].

COVID-19-associated microthrombosis is initiated via the endothelial exocytosis of ultra-large von Willebrand factor multimeric glycoproteins and antihemophilic globulins A from the Weibel–Palade bodies. If the ADAMTS-13 levels are insufficient to cleave the large vWF multimers, the latter will activate intravascular thrombosis by anchoring to the damaged endothelial cells and recruiting platelets, triggering the formation of “microthrombi strings” [17]. The generation of antibodies against ADAMTS13 appears to be a frequent and unique finding in COVID-19, supporting “COVID-19 immunothrombosis”, a term coined recently to embed both micro- and macrovascular thrombotic events associated with the disease [18].

COVID-19-associated pulmonary thrombosis is an in situ immunothrombosis not related to venous thromboembolism, in which similar mechanisms involving endothelial injury and a loss of anticoagulant properties can be incriminated [19].

It has also been proposed that the virus may trigger complement activation by acting as a cofactor to enhance lectin pathway activation [20]. The terminal complement complex C5b-C9 (MAC—membrane attack complex) causes the formation of pores on the membranes of the endothelial cells leading to endothelial damage when the MAC-inhibitor CD59 is under-expressed and cannot properly exhibit its regulatory function [21]. C5a can stimulate the release of TF and plasminogen activator inhibitor-1 (PAI-1) and activate neutrophils, which release cytokines and neutrophil extracellular traps (NETs). NETs are structures of DNA, histones, and antimicrobial proteins that bind and kill pathogens. The excessive production of NETs can facilitate microthrombosis by creating a scaffold for platelet aggregation, thus contributing to the vicious pro-thrombotic circle [22].

The link between coagulopathy in viral infections and COVID-19 is discussed in a recent review published in a Special Issue by Ragnoli et al. [23]. Two possible mechanisms implicated in the pathogenesis of coagulation dysfunction during SARS-CoV2 infection are reviewed here: the cytokine storm and virus-specific mechanisms related to the virus interaction with the renin–angiotensin system and the fibrinolytic pathway. The role of IL-1 and IL-6, as well as IL-18 is emphasized. Moreover, a reduction in endothelial nitric oxide synthase activity and nitric oxide levels is cited as a possible pathogenic culprit of endothelial dysfunction. They also discuss the very interesting topic of thrombocytopenia induced by COVID-19 vaccination. In rare cases, the immune thrombotic thrombocytopenia (VITT) syndrome was induced by the vaccine, particularly by the ChAdOx1 nCoV-19 vaccine (COVID-19 Vaccine AstraZeneca) [23]. The hypothesis is the possible recruiting of antibodies against platelet factor 4 (PF4), inducing massive platelet activation and immune thrombotic thrombocytopenia [24].

SARS-CoV-2 penetration into the cells is dependent on glycans with sialic acid (SA) terminal moieties found on the viral spike protein (SP), which serve as the initial attachment anchors to red blood cells (RBCs), platelets, leukocytes, and endothelial cells. Hemagglutination is a defense mechanism used by RBCs and platelets against pathogens expressing SA terminal moieties, involving pathogen attachment, followed by delivery to leukocytes for phagocytosis, in a process termed “immune adherence”. The capacity of this initial defense mechanism is surpassed in severe COVID-19 infections, leading to high levels and sizes of RBC rouleaux (stacked clumps) exceeding the leukocyte capacity to sequester them, as discussed in the review by Sheim et al. [25]. The risk factors of increased age, diabetes, and obesity associated with COVID-19 were found to also be associated with significantly increased RBC aggregation, another valuable conclusion by Sheim et al. [25]. SARS-CoV-2 SP attachments to RBCs were demonstrated directly by Lam et al. [26] through immunofluorescence imaging. In this Special Issue, Boschi et al. [27] show that SARS-CoV-2 SP from various strains induced hemagglutination when mixed with human RBCs. These results are in line with other studies documenting associations between RBC aggregation and microvascular occlusion in severe COVID-19 [28,29].

Microthrombi in the heart, kidneys, and liver were also frequently observed in autopsy examinations of COVID-19 patients, suggesting that these may have contributed to multiorgan damage and failure. A report from Koutsiaris et al. [30] demonstrated the persistence of microthrombosis even after recovery, as demonstrated by video capillaroscopy of the ocular microvessels of severe COVID-19 patients within 28 days post-discharge.

Wada et al. [31] reviewed some of the most studied mechanisms of thrombosis as important factors leading to a negative COVID-19 patient evolution: old age, long-time bed rest and comorbidities, inflammation, cytokine storms, vascular endothelial injuries, PTE, hypoventilation, a hypercoagulable state (including activation of the TF pathway), NETs, hypofibrinolysis, and platelet activation. The authors illustratively compare the mechanism underlying thrombosis in COVID-19 and bacterial infections.

The molecular mechanisms of direct and indirect effects of the spike protein on the expression of adhesion molecules, markers of endothelial injury, and elevated inflammation are presented in the work of Bhargavan and Kanmogne [32]. According to Wada et al. [31], among the most valuable routine biomarkers for the evaluation of thrombosis in COVID-19 are CWA-APTT (clot waveform analysis of activated partial thromboplastin time) and TF-induced factor IX activation assay (sTF/FIXa). Although D-dimer is useful for the exclusion of VTE in COVID-19 patients, it is too unspecific for VTE diagnosis, as its cut-off level is low in these patients.

Soluble platelet membrane glycoprotein VI (sGPVI) and soluble C-type lectin-like receptor 2 (sCLEC-2) were also proposed as platelet activation biomarkers [33]. The presence of activated platelets causing severe microangiopathy in patients with COVID-19 may be detected by the release of large amounts of sCLEC-2 into the blood [33]. Studies have also detected a mild decrease in ADAMTS-13 in plasma, but the clinical significance of this finding remains unclear [34].

Increased fibrinogen and PAI-I levels have been found to be the biomarkers of hypo-fibrinolysis, reducing the capacity to dissociate thrombi. In advanced COVID-19 patients, vascular endothelial cell injury markers such as soluble thrombomodulin (sTM), VWF, and PAI-I are high, while AT (antithrombin) levels are low [35]. It is thought that the ensuing hypo-fibrinolysis may contribute to organ failure in these patients [35].

The early recognition of a hyperinflammatory and hypercoagulation state would allow for the timely application of preventive measures against a fulminant disease evolution. In their study, Făgărașan et al. [36] demonstrated that IL-6 and the neutrophil–lymphocyte ratio (NLR) predicted disease severity in COVID-19 patients with diabetes mellitus (DM). Significant associations between IL-6 levels and disease evolution have also been described in non-diabetics [37]. Further studies are needed to elucidate the role of IL-6 in this context to determine the cut-off values associated with worse outcomes and explore the potential of IL-6 as a treatment target in COVID-19 [37].

A number of studies emphasized the procoagulant profile of COVID-19 patients during the acute phase of the illness, but less is known about the short- and long-term effects. The long-term persistence of COVID-19-related coagulopathy, along with long-lasting lung dysfunction, have been noted since the beginning of the pandemic [38,39,40].

Of particular interest for this Special Issue is the anti-coagulant therapy in COVID-19. As the pathogenesis of SARS-CoV-2-induced coagulopathy is incompletely understood and multifactorial, the use of antithrombotic therapy is difficult to standardize. Three pivotal phase III randomized clinical trials regarding antithrombotic agents were conducted, starting from 2020: INSPIRATION, Remap/cap/ACTIV-4a/ATTACC, and RECOVERY INSPIRATION Investigators [41,42,43]. The studies showed a lack of improvement in the outcome of critically ill patients receiving intermediate-dose prophylactic anticoagulants. This outlined the superiority of the therapeutic anticoagulant dose compared to the prophylactic dose for the survival of non-critically ill patients, but not in the critically ill ones, and the failure of Aspirin to reduce the 28-day mortality or progression to mechanical ventilation/death in hospitalized patients [42]. Similarly, the ACTIVE-4a trial showed that the use of P2Y12 receptor inhibitors did not improve the number of organ support-free days in patients with mild forms of COVID-19 [44]. Importantly, the use of antithrombotic therapies requires the careful balancing of thrombotic and bleeding risks. The measurement of serum AT-III activity is recommended in the algorithm of evaluating SARS-CoV-2-infected patients, as emphasized by the review of Szilveszter et al. [45].

According to guidelines, a prophylactic or therapeutic dose of low-molecular-weight heparin (LMWH) should be administered to all patients as prophylaxis against venous thromboembolism (VTE) and PTE, particularly in those with a high thrombosis risk (that is, patients with elevated D-dimer levels) and a low bleeding risk [46]. For patients who are transferred to an intensive care unit, increasing from a prophylactic to a therapeutic LMWH dose is recommended. Patients with heparin resistance caused by AT-III deficiency may be treated with direct thrombin inhibitors, such as Argatroban. The use of oral anticoagulants is not recommended in COVID-19 [46]. Considering the substantial contribution of inflammation to COVID-19-associated coagulopathy, the development of anti-inflammatory therapies to treat COVID-19 might interfere with anticoagulation, raising the risk of bleeding. The combined use of anti-inflammatory and anticoagulant drugs must be evaluated in further clinical trials [47].

As underlined by the studies discussed above, we are happy to conclude that the Special Issue “COVID-19 Coagulopathy: Advances in Pathophysiology and Therapies” has brought important contributions to understand the underlying mechanisms of SARS-CoV-2-induced coagulopathy to define diagnostic and prognostic biomarkers for COVID-19 patients, and discuss the potential anti-coagulant and anti-thrombotic therapies in this complex disease.
